# Ultrastructural and cytochemical aspects of female gametophyte development in *Sedum hispanicum* L. (Crassulaceae)

**DOI:** 10.1007/s00709-017-1155-3

**Published:** 2017-08-24

**Authors:** Emilia Brzezicka, Małgorzata Kozieradzka-Kiszkurno

**Affiliations:** Department of Plant Cytology and Embryology, Faculty of Biology, University of Gdańsk, 59 Wita Stwosza Street, 80-308 Gdańsk, Poland

**Keywords:** Embryo sac, Megagametogenesis, Megasporogenesis, Plasmodesmata, Polygonum type, Ultrastructure

## Abstract

Until now, development of the female gametophyte has been investigated only in some species of Crassulaceae using a light microscope. To the best of our knowledge, this is the first report that describes the process of megasporogenesis and megagametogenesis in Crassulaceae in detail. To achieve this, we performed embryological studies on *Sedum hispanicum* L. (Crassulaceae). Cytochemical analysis detected the presence of proteins, lipids, and insoluble polysaccharides in individual cells of the gametophyte. The development of the embryo sac conforms to the monosporic or *Polygonum*-type in anatropous, crassinucellate, and bitegmic ovules. One megaspore mother cell initiates the process of megasporogenesis. Prior to the first meiotic division, the nucleus is centrally located within the meiocyte. Other organelles seem to be distributed evenly over the micropylar and chalazal parts during the development. Most storage reserves detected during megasporogenesis were observed in the megaspore mother cell. Three mitotic divisions within the chalazal functional megaspore resulted in the enlargement of the eight-nucleated embryo sac. In the seven-celled gametophyte, three chalazally located antipodes degenerated. A mature embryo sac was formed by the egg apparatus and central cell. When the antipodes degenerated, both synergids became organelle-rich and more active. The concentration of lipid droplets, starch grains, and proteins increased during megagametogenesis in the growing gametophyte. In the cellular embryo sac, the central cell can be distinguished by its largest accumulation. Our data confirm the hypothesis that plasmodesmata with electron-dense dome are formed during development of the female gametophyte in *S. hispanicum* and not just during the stages of embryogenesis. We observed these structures in megaspores and coenocytic embryo sac walls. Functions of observed plasmodesmata are discussed.

## Introduction

The life cycle of seed plants can be distinguished into two generations—gametophyte and sporophyte. The female gametophyte develops within the ovule, which is located in the ovary formed by the carpel(s). This is followed by megasporogenesis and megagametogenesis, which then forms an embryo sac (Yadegari and Drews 2004; Lituiev and Grossniklaus 2014). The first process, which is megasporogenesis, is associated with the production of the functional megaspore from megaspore mother cell after meiosis. The second process, which is megagametogenesis, continues, resulting in the formation of the mature embryo sac after mitotic division(s) in the megaspore. The process of cellularization is necessary to form the cellular gametophyte (Willemse and Van Went 1984; Russell 2001; Ma and Sundaresan 2010).

The Crassulaceae comprises about 1400 species in 33–35 genera (Eggli 2003; Christenhusz and Byng 2016)*.* Most of their representatives have succulent leaves, but within Crassulaceae, plants with succulent stems and with caudices can also be found (Eggli 2003). Embryological studies on members of Crassulaceae have been previously performed, but their current information is scarce. The structure and process of formation of the embryo sac (megasporogenesis and megagametogenesis) have been investigated in some Crassulaceae species by using a light microscope (Sharp 1913; Souéges 1927; Mauritzon 1933; Johri et al. 1992; Wojciechowicz and Samardakiewicz 1998; Thiede and Eggli 2007). In Crassulaceae, the female gametophyte develops in an anatropous ovule, in which the nucellus is covered with the outer and inner integuments (bitegmic). Three different types of nucelli have been distinguished: *Sedum*, *Crassula*, and *Kalanchoe* (Mauritzon 1933). This classification is used only for representatives of Crassulaceae (Wojciechowicz and Samardakiewicz 1998).

*Sedum hispanicum* L. belongs to a speciose genus *Sedum* L. (ca. 420 species) (Thiede and Eggli 2007) within Crassulaceae, which is simultaneously highly polyphyletic (Nikulin et al. 2016). Previously published data are mainly schematic drawings made from microscopic observations, which also show some stages of megagametogenesis in *S. hispanicum*. They suggest that the development of the gametophyte in *S. hispanicum* is of the *Polygonum*-type (Batygina and Yakovlev 1985; Johri et al. 1992). In Crassulaceae, the monosporic *Polygonum*-type is the most frequent type of female gametophyte development observed (Mauritzon 1933; Thiede and Eggli 2007). *Polygonum*-type female gametophyte development has also been observed in 70–80% of the angiosperms and has been described as the normal type of development (Maheshwari 1950; Johri et al. 2001; Yadegari and Drews 2004). It means that the haploid, one-nucleate, chalazal megaspore forms a seven-celled embryo sac after three mitotic divisions and gametophyte cellularization (Johri et al. 2001; Lersten 2004; González-Gutiérrez et al. 2014).

*Scilla*-type embryo sac development has been found in some representatives of the genus *Sedum* (Mauritzon 1933). This type of development has also been described as the *Endymion*-type (Rodkiewicz 1973); however, drawings published by Mauritzon (1933) conform it as the *Allium*-type. Other authors have reported similar contradictions (Herr 1984), but most of the data indicate that it is the *Allium*-type of development. A bisporic-type embryo sac is present in *Sedum fabaria*, *S. populifolium*, and *S. populifolium* var. *notarjanni* (Mauritzon 1933; Wojciechowicz and Samardakiewicz 1998). The mature female gametophyte is formed after two mitotic divisions and the process of cellularization of the coenocytic embryo sac (Maheshwari 1950). Its final structure is similar to that of a normal *Polygonum-*type embryo sac. Moreover, haustoria are observed in a few species of the genus *Sedum*. Haustoria can form during megasporogenesis and/or megagametogenesis in gametophytes by *Polygonum*- and an *Allium*-type embryo sacs (Mauritzon 1933). The simultaneous formation of haustorium and *Allium*-type female gametophyte has been observed only within the representatives of Crassulaceae belonging to genus *Sedum*.

Until now, ultrastructural and cytochemical studies of Crassulaceae ovules have been conducted on samples in which the process of fertilization has occurred. In addition, researchers have focused on the structure and development of the embryo (including on the haustorial suspensor) (Kozieradzka-Kiszkurno and Bohdanowicz 2006; Kozieradzka-Kiszkurno et al. 2012; Czaplejewicz and Kozieradzka-Kiszkurno 2013). Therefore, further research on ultrastructural and cytochemical aspects of embryo sac development is highly necessary to advance our knowledge regarding the family. Moreover, some of the previous studies have reported on the ultrastructure of the branched plasmodesmata with electron-dense dome (Kozieradzka-Kiszkurno and Bohdanowicz 2010; Kozieradzka-Kiszkurno et al. 2011; Kozieradzka-Kiszkurno and Płachno 2012). Such structures possibly are present during other stages of embryological development in Crassulaceae. In this article, we tested the hypothesis that in *S. hispanicum*, during megasporogenesis and megagametogenesis, characteristic plasmodesmata with electron-dense dome are formed.

The primary aim of this study was to describe in detail the process of megasporogenesis and megagametogenesis in *S. hispanicum* L. (Crassulaceae). To the best of our knowledge, this is the first report on the ultrastructural and cytochemical analyses of female gametophyte of *S. hispanicum*. Furthermore, we also discuss the observations in light of the currently available knowledge for species of angiosperms.

## Materials and methods

### Plant material

In this study, ovules of *S. hispanicum* L. at different developmental stages isolated from flower buds as well as flowers after anthesis were studied. Plants of *S. hispanicum* were obtained from their natural habitat in Gdańsk (northern Poland). Materials for microscopic examination were collected from May to June in the years 2015 and 2016.

### Electron microscopy

Ovules of *S. hispanicum* were isolated from flower buds and were fixed in 2.5% glutaraldehyde and 2.5% formaldehyde (prepared from paraformaldehyde) in 0.05 M cacodylate buffer (pH = 7.0) for 4 h at room temperature. The fixed ovules were rinsed in cacodylate buffer and treated with 1% osmium tetroxide in cacodylate buffer (overnight at 4 °C). Then, the specimens were washed (cacodylate buffer and distilled water) and treated with 1% uranyl acetate in distilled water for 1 h. Next, the specimens were rinsed in distilled water, dehydrated in an increasing acetone series, and embedded in Spurr’s epoxy resin (Spurr 1969). The specimens were cut with a diamond knife on a Leica EM UC7 ultramicrotome. Ultrathin sections (50–100 nm) were post-stained with a saturated solution of uranyl acetate in 50% ethanol and 0.04% lead citrate. Finally, samples were examined using a Philips CM 100 and FEI Tecnai G^2^ Spirit TWIN/BioTWIN transmission electron microscope in the Faculty of Biology, University of Gdańsk (Poland).

### Light microscopy

For light microscopy, semi-thin sections (0.5–1.5 μm) were cut with a glass knife on Sorvall MT 2B ultramicrotome from the specimens embedded in Spurr’s resin. Then, the sections were stained with 0.05% toluidine blue O in 1% sodium tetraborate. For detection of insoluble polysaccharides, proteins and lipids were stained with the periodic acid-Schiff (PAS) reagent (Jensen 1962), aniline blue black (ABB; Jensen 1962), and with Sudan black B (SBB; Bronner 1975). The microscopical analysis and photographic documentation were made with a Nikon Eclipse E 800 light microscope and a Nikon DS-5Mc camera using the Lucia Image software.

For control, the ovules at different developmental stages were cleared in Hoyer’s solution (Liu and Meinke 1998). First, the plant material was fixed in methanol:acetic acid (3:1) and then washed in decreasing concentrations of methanol (96, 75, and 50%) for 30 min. The material was gradually cleared in Hoyer’s solution. Finally, the samples were examined with Nikon Eclipse E 800 microscope with differential interference contrast optics (DIC).

## Results

**Fig. 1:**
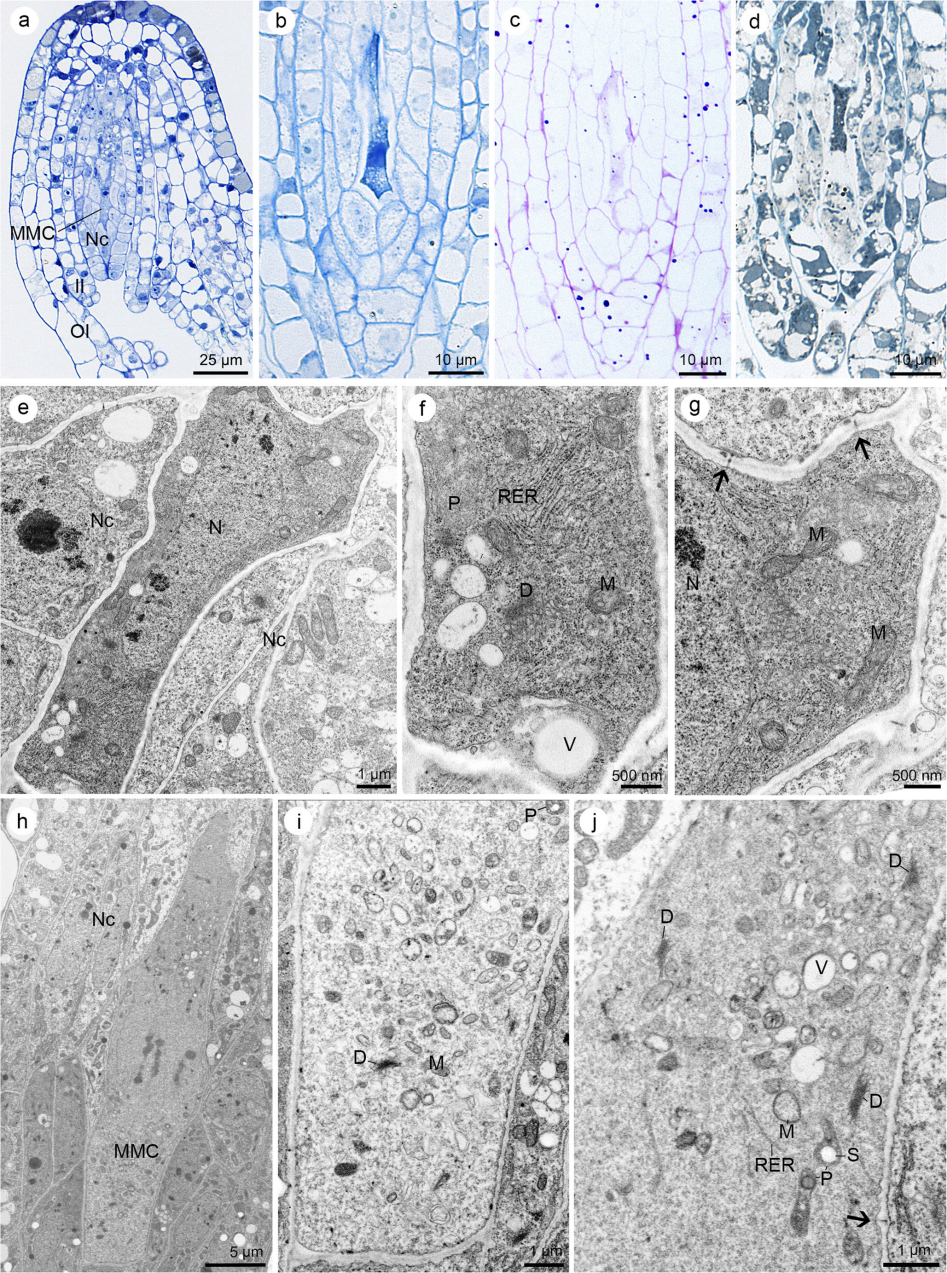
Ovule of *Sedum hispanicum* at megaspore mother cell (MMC) stage. **a**–**d** Light micrographs—results of cytochemical tests. **e**–**j** Electron micrographs. **a** Longitudinal section showing the MMC located within the anatropous, crassinucellate ovule. Outer and inner integuments (OI and II, respectively) cover the nucellus, nucellar cells (Nc). **b** The MMC stains intensively with aniline blue black, which suggests the occurrence of proteins. **c** Semi-thin section stained with periodic acid-Schiff (PAS) reagent. The MMC shows the presence of starch grains. **d** Section stained with Sudan black B showing the presence of lipid droplets in megasporocyte. **e** Slightly elongated MMC contains electron-dense cytoplasm in comparison with Nc. The conspicuous nucleus (N) occupies the most portion of the cell. **f** Micropylar region of the megasporocyte with visible plastid (P), cisternae of rough endoplasmic reticulum (RER), mitochondria (M), single dictyosomes (D), and small vacuoles (V). **g** Plasmodesmata cross the chalazal walls of the MMC (arrows). Mitochondria (M) and nucleus (N) are visible in the electron-dense cytoplasm. **h** Strongly elongated MMC during the first meiotic division with visible neighboring Nc. Its cytoplasm is less electron-dense in comparison with an earlier stage (**e**). **i** View of the micropylar part of the megasporocyte. Dictyosomes (D), mitochondria (M), and plastids (P) with starch grains occur in cytoplasm. **j** Chalazal portion of the MMC with visible plasmodesmata (arrow) in the cell wall. Cytoplasm contains dictyosomes (D), mitochondria (M), plastids (P) with starch grains (S), small vacuoles (V), and rough endoplasmic reticulum (RER)

The ovules of *S. hispanicum* L. are anatropous and crassinucellate, and the micropyle is formed by outer and inner integuments (bitegmic) as in other Crassulaceae (Fig. 1a). The development of the female gametophyte is monosporic or *Polygonum*-type, and during this process, the formation of haustoria was not observed. Cytochemical staining revealed the presence of proteins, lipids, and insoluble polysaccharides, both in the developing embryo sac and other ovular tissue.

### Megasporogenesis

The process of megasporogenesis is initiated with one, mononuclear megasporocyte (Fig. 1a–g). The megasporocyte is formed within the ovule, which is not fully mature. At this stage, the nucellus is not completely covered with integuments. At the micropylar end of the nucellus, the meiocyte is located, which is separated from the nucellar epidermis by two layers of cells (Fig. 1a). At the beginning, the megaspore mother cell differs slightly from the surrounding nucellar cells, but during the development, it becomes more elongated and enlarged. Megasporocyte shows a strong reaction to the proteins (Fig. 1b). Cytochemical staining also showed the presence of insoluble polysaccharides (Fig. 1c) and lipids (fine droplets within cytoplasm) (Fig. 1d). The above data were confirmed by ultrastructural observations. The young megaspore mother cell differs from the surrounding nucellar cells with respect to size, shape, and a dense cytoplasm. The nucleus is elongated and occupies a large part of the cell (Fig. 1e). Within the cytoplasm, the abundant cisterns of rough endoplasmic reticulum (RER), plastids, mitochondria, single dictyosomes, and small vacuoles are visible (Fig. 1f, g). Plasmodesmata are present in chalazal walls of the megasporocyte (Fig. 1g). The megaspore mother cell elongates considerably during its development (Fig. 1h). Prior to the first meiotic division, its nucleus is located centrally within the cell. At this stage, plastids with starch grains, dictyosomes, small vacuoles, and mitochondria are also visible in the cytoplasm (Fig. 1i, j).

**Fig. 2:**
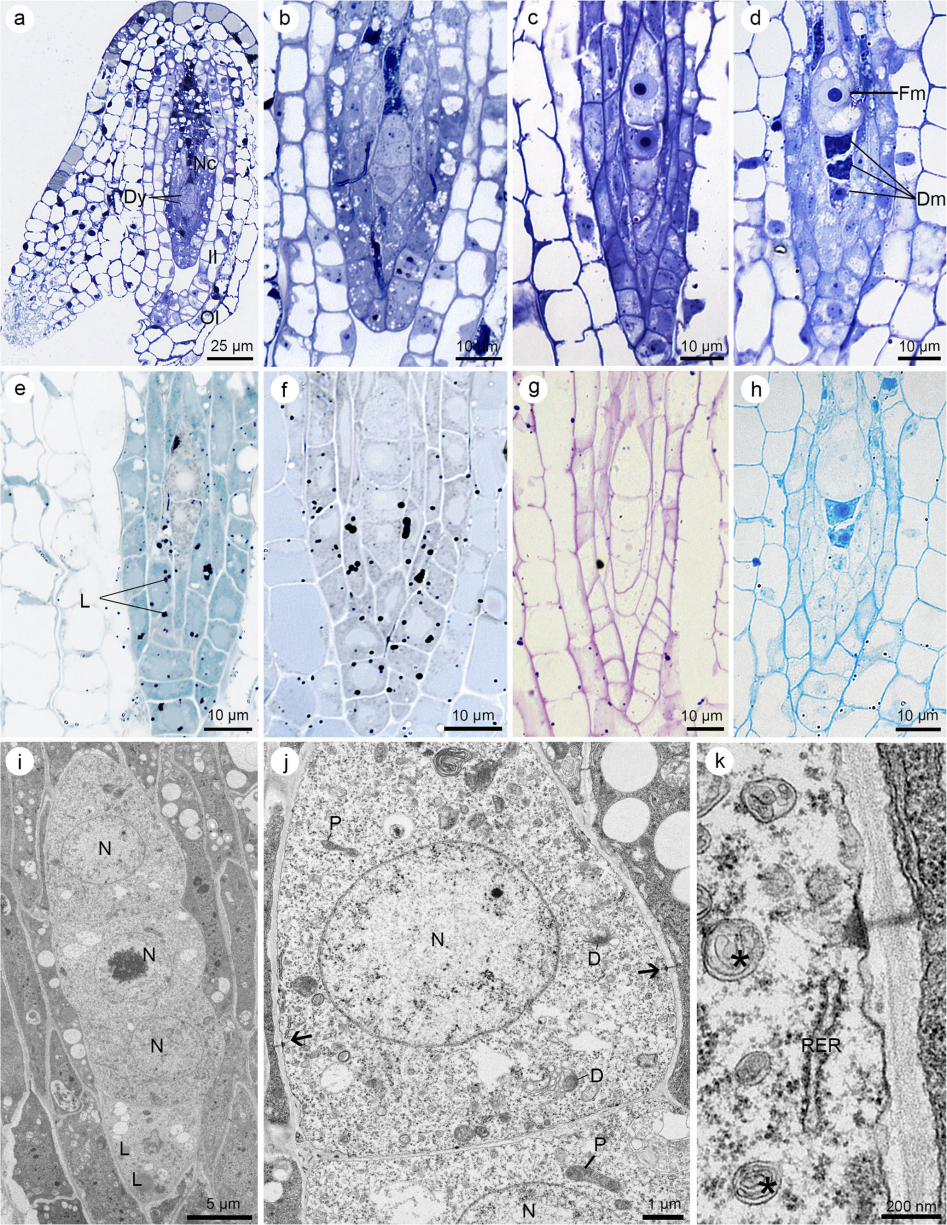
Dyad and tetrad stages of the female gametophyte development in *Sedum hispanicum*. **a**–**h** Light micrographs—results of cytochemical tests. **i**–**k** Electron micrographs. **a** General structure of the ovule at the dyad (Dy) stage. Nucellar cells (Nc) and outer and inner integuments (OI and II, respectively) are visible. **b** Higher magnification of the dyad from **a**, which consists of two uninucleate cells with a similar size. **c** Linear tetrad of megaspores. Chalazal cell is the biggest one. **d** Formation of functional megaspore (Fm). Three micropylar megaspores degenerate (Dm). **e** Sudan black B staining reveals the lipid droplets (L) localization at the dyad stage. **f** Semi-thin section showing the lipids’ occurrence at the tetrad stage after Sudan black B staining. **g** Distribution of the polysaccharides at the tetrad stage visible after periodic acid-Schiff (PAS) reaction. **h** Section stained with aniline blue black shows that degenerating megaspores stain more intensely for proteins than functional megaspore. **i** View of the linear tetrad. Lipid droplets’ (L) occurrence is confirmed ultrastructurally. All megaspores are uninucleate (N). **j** Higher magnification of chalazal megaspore from **i**. Cytoplasm contains one nucleus (N), dictyosomes (D), and plastids (P). Simple plasmodesmata with adjacent electron-dense material from the megaspore cell end (arrows) cross the walls of the megaspore tetrad. **k** Details of the lateral wall of chalazal megaspore from **j** with visible multilamellar bodies (asterisks) in the cytoplasm. The RER occurs near the simple plasmodesma with electron-dense dome

In the megasporocyte, the first and second meiotic divisions occur. Then, the cell walls are formed; thus, the dyad (Fig. 2a, b) and tetrad (Fig. 2c, d) stages appear. A dyad consists of two mononuclear cells of uniform size with accumulated lipid droplets (Fig. 2e) and starch grains. The result of meiotic division and cytokinesis is one linear tetrad situated along the micropylar–chalazal axis. Chalazal megaspore appears to be the largest cell of the tetrad. Lipid storage is confirmed in all mononuclear cells (Fig. 2f). Simultaneously, four megaspores give a weak reaction for insoluble polysaccharides (Fig. 2g) and proteins. The final stage of *S. hispanicum* megasporogenesis is the formation of a functional megaspore (chalazal cell) (Fig. 2d). Three micropylar megaspores degenerate and stain intensively with ABB (Fig. 2h).

All cells of the linear tetrad have few lipid droplets (Fig. 2i), which is also compatible with cytochemical observations (Fig. 2f), active dictyosomes, plastids, mitochondria, profiles of RER, and multilamellar bodies (Fig. 2j, k). Walls of *S. hispanicum* megaspores show the presence of simple plasmodesmata with adjacent electron-dense dome from the megaspore cytoplasm side (Fig. 2j, k). These structures are also observed in the functional megaspore (not shown). ER occurs near these characteristic plasmodesmata (Fig. 2k).

### Megagametogenesis

**Fig. 3:**
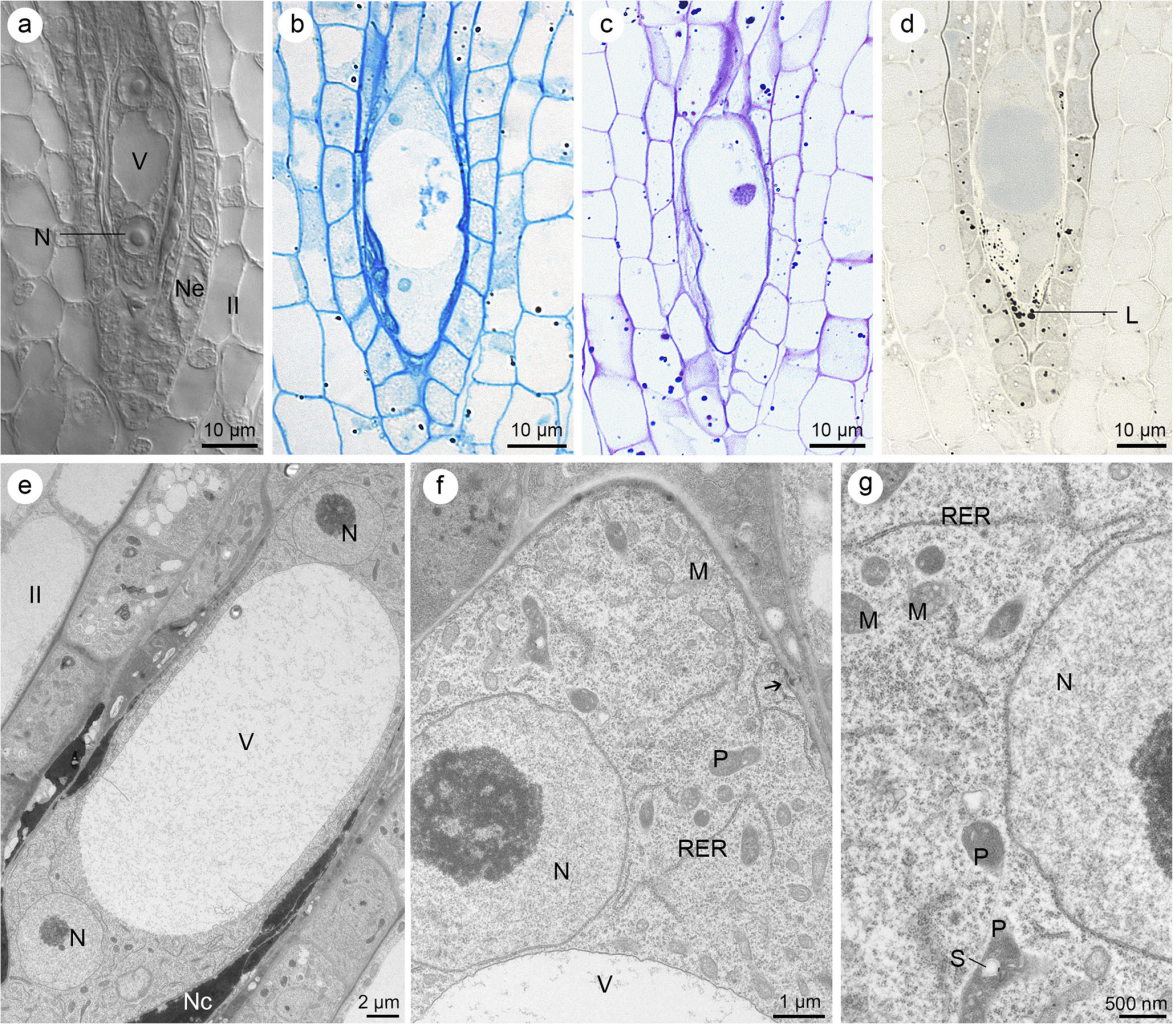
Results of mitotic divisions, without cytokinesis of the functional megaspore. **a** DIC image. **b**–**d** Light micrographs—results of cytochemical tests. **e**–**g** Electron micrographs. **a** Cleared ovule showing the two-nucleate embryo sac; nucleus (N), vacuole (V), nucellar epidermis (Ne), and inner integument (II) are visible. **b** Four-nucleate gametophyte stained with aniline blue black contains a few proteins. **c** Periodic acid-Schiff (PAS) reaction shows the localization of insoluble polysaccharides in four-nucleate gametophyte (the same ovule as in **b**). **d** Lipid (L) distribution at the four-nucleate embryo sac visible after Sudan black B staining. **e** Ultrastructure of two-nucleate gametophyte with nuclei (N) and large vacuole (V). Degenerating neighboring nucellar cells (Nc) and fragments of inner integument (II) are visible. **f** Chalazal part of the embryo sac from **e** with visible nucleus (N), plastids (P), profiles of rough endoplasmic reticulum (RER), vacuole (V), mitochondria (M), and plasmodesmata with adjacent electron-dense dome (arrow). **g** Higher magnification of cytoplasm from the micropylar end of the two-nucleate embryo sac (**e**) showing the nucleus (N), profiles of RER, mitochondria (M), and plastids (P) with starch grain (S)

The megagametogenesis is initiated by three mitotic divisions (without cytokinesis) of the functional megaspore. After the first mitotic division, nuclei move to the opposite poles of the cell (Fig. 3a). Following the mitotic cycle, four- and eight-nucleate embryo sacs are formed. A large central vacuole occupies the middle part of the developing female gametophyte (Fig. 3a–e). Sometimes at the chalazal end, a small-sized vacuole is also formed (not shown). Cytochemical tests reveal that the cytoplasm of the multinucleate embryo sac contains a few proteins (Fig. 3b), insoluble polysaccharides (Fig. 3c), and lipids (mainly localized at the micropylar part of the gametophyte) (Fig. 3d). The embryo sac expands and destroys the surrounding nucellar cells (Fig. 3e). Plasmodesmata with electron-dense dome occur in the walls of the coenocytic embryo sac too (Fig. 3f). The central vacuole pushes to the outside of a cytoplasm, in which cisterns of RER, mitochondria, and plastids with starch grains can be distinguished (Fig. 3f, g).

**Fig. 4:**
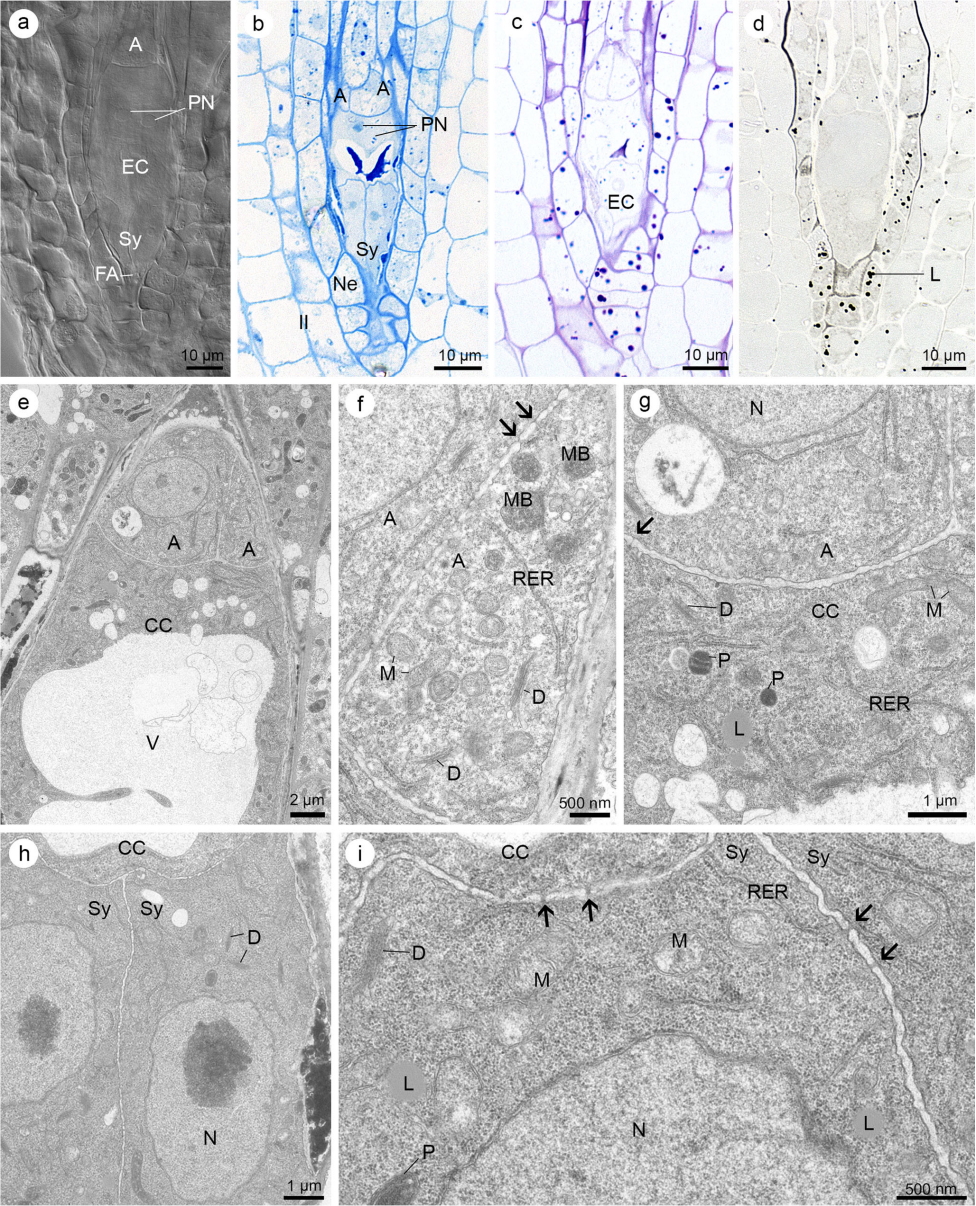
Seven-celled embryo sac of *Sedum hispanicum.* **a** DIC image. **b**–**d** Light micrographs—results of cytochemical tests. **e**–**i** Electron micrographs. **a** Cleared ovule showing the seven-celled embryo sac: three antipodes (A), central cell with two polar nuclei (PN) located at the chalazal end of the cell, egg cell (EC), and two synergids (Sy) with filiform apparatus (FA). **b** Section stained with aniline blue black reveals the localization of proteins within the cellular embryo sac: antipodes (A), polar nuclei (PN) located at the chalazal end of the cell, and synergids (Sy). Nucellar epidermis (Ne) and inner integument (II) are visible. **c** Distribution of the polysaccharides within the embryo sac cells revealed after staining with periodic acid-Schiff (PAS) reagent, egg cell (EC). **d** Lipid droplets (L) distribution visible after Sudan black B staining. **e** Structure of the chalazal end of the embryo sac with antipodes (A), central cell (CC) with a big central vacuole (V). **f** Details of the antipodes (A) filled with cytoplasm containing microbodies (MB) with adjoining rough endoplasmic reticulum (RER), numerous mitochondria (M), and same active dictyosomes (D). Plasmodesmata occur in walls between antipodal cells (arrows). **g** Fragments of the antipode (A) and central cell (CC) with visible plasmodesmata (arrow) separating the cell wall. Cytoplasm of central cell is rich in organelles: dictyosomes (D), mitochondria (M), plastids (P), and profiles of RER. The presence of lipid droplets (L) is also confirmed ultrastructurally. Fragment of nucleus is visible (N). **h** Micropylar part of the embryo sac with central cell (CC) and two, uninucleate synergids (Sy). Nucleus (N) is located in the middle part of the synergid. Some dictyosomes (D) are also present in the cytoplasm. **i** Higher magnification of synergid cytoplasm with noticeable lipids (L), nucleus (N), plastids (P), mitochondria (M), few dictyosomes (D), and profiles of RER. Cell walls between two synergids (Sy) and synergids and central cell (CC) are perforated by plasmodesmata (arrows)

**Fig. 5:**
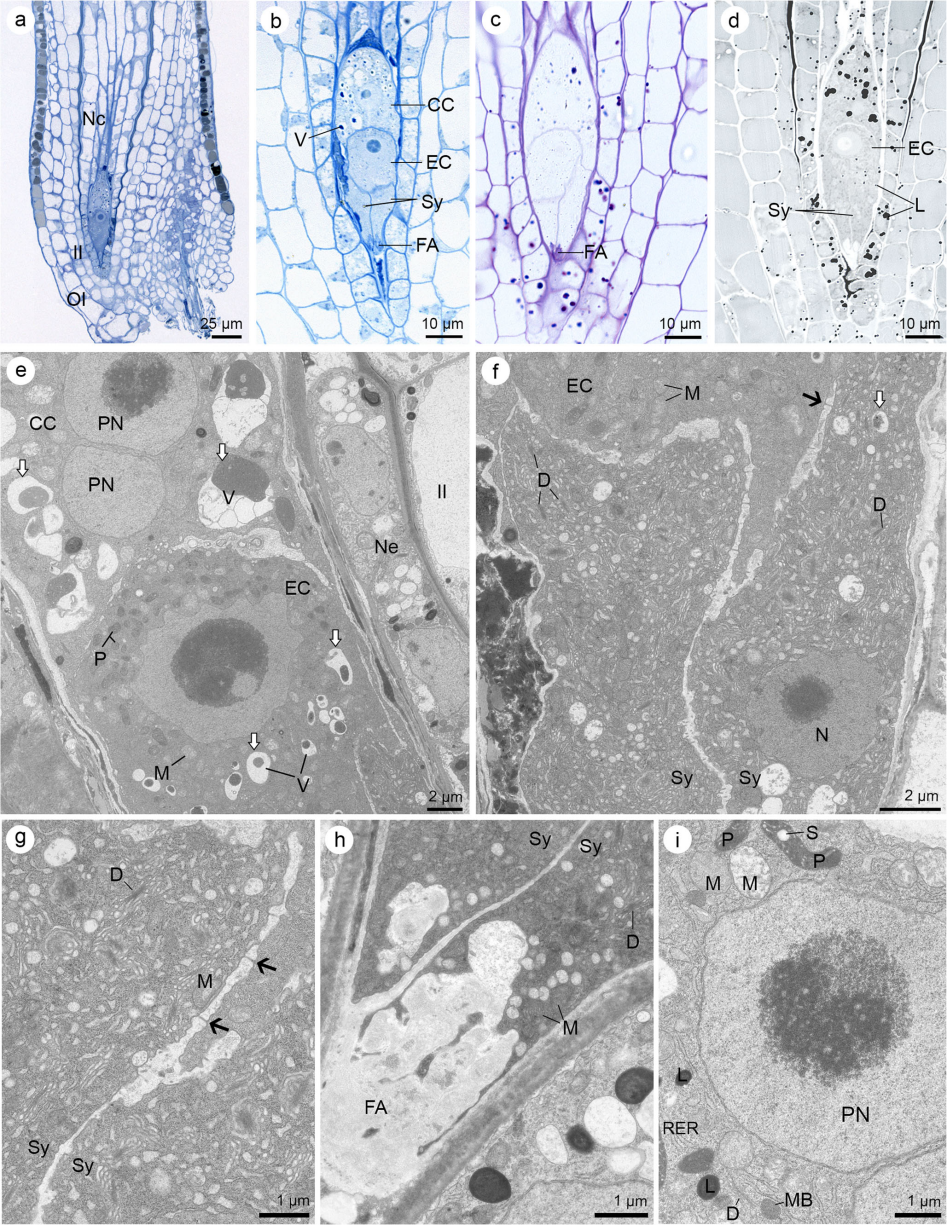
Mature female gametophyte of *Sedum hispanicum*. **a**–**d** Light micrographs—results of cytochemical tests. **e**–**i** Electron micrographs. **a** Longitudinal section of the anatropous ovule with visible mature embryo sac, nucellar cells (Nc), and inner and outer integuments (II and OI, respectively). **b** Semi-thin section stained with aniline blue black. Four-celled embryo sac consists of: central cell (CC) with vacuoles (V) containing protein material (it stains intensively with aniline blue black (ABB)), egg cell (EC), and synergids (Sy) with filiform apparatus (FA), which stains clearly with ABB. **c** Localization of insoluble polysaccharides detectable after PAS staining. FA is built of PAS-positive ingrowths, which occur in the micropylar part of the synergids. **d** Detection of lipid droplets (L) with Sudan black B. In the central cell, the lipids’ occurrence is greater than that in the egg cell (EC) and synergids (Sy). **e** Detailed structure of the central cell (CC) and egg cell (EC). Polar nuclei (PN) of the central cell adhere to each other and occur near the egg cell. Note the vacuoles (V) with electron-dense material within the central cell and the egg cell. These macroautophagous structures are indicated by white arrows. Plastids (P) are visible at the chalazal part of the egg cell, near the cell wall, which thin out locally. Some mitochondria (M) are also present in the egg cell cytoplasm. Nucellar epidermis (Ne) and fragments of inner integument (II) are visible. **f** Chalazal end of the synergids (Sy) with visible nucleus (N). Plasmodesmata (arrow) occur in discontinuous cell wall between synergids and egg cell (EC). Thinning of synergids’ cell wall is visible. Note the mitochondria (M) and active dictyosomes (D) and macroautophagous structures (white arrow). **g** Higher magnification of synergids (Sy) from (f) showing numerous dictyosomes (D), mitochondria (M), and plasmodesmata in the cell wall (arrows). Cell wall is clearly discontinuous. **h** Micropylar apex of synergids (Sy) with visible electron-translucent filiform apparatus (FA). Mitochondria (M) and active dictyosomes (D) are located close to the wall ingrowths. **i** Portion of the central cell showing the polar nucleus (PN), plastids (P) with starch grain (S), lipid droplets (L), dictyosomes (D), mitochondria (M), profiles of rough endoplasmic reticulum (RER), and microbodies (MB)

The result of the cell wall formation is a seven-celled embryo sac (Fig. 4a). Egg apparatus (two synergids and one egg cell) is located at its micropylar end, whereas three antipodes are formed at the chalazal end. The binucleate central cell occupies the largest middle part of the embryo sac. First, the polar nuclei are arranged closely with respect to chalaza (Fig. 4a, b), but they change their location during the development of the gametophyte. They approach each other and are arranged close to the egg cell (Fig. 5e). Antipodes and the remaining cells of the embryo sac stained positive with ABB and PAS tests for the detection of proteins (Fig. 4b) and insoluble polysaccharides, respectively (Fig. 4c). Low lipid accumulation was observed in antipodal cells (Fig. 4d). At this stage, the egg and the central cell have clearly visible starch grains (Fig. 4c). The central cell (Fig. 4b, d) and the egg cell (not shown at this developmental stage), stained with ABB and SBB, respectively, which suggests the occurrence of proteins and lipids in these cells.

Each of the antipodal cell has a single nucleus, mitochondria, dictyosomes, and microbodies. Microbodies are in close contact with the ER (Fig. 4e, f). At this stage, the central cell is highly vacuolated (Fig. 4e). Cytoplasm is limited to a thin layer. It is characterized by the presence of ER and lipid droplets (which was earlier confirmed cytochemically), plastids, dictyosomes, and mitochondria (Fig. 4g). Synergids are located at the micropylar region of the embryo sac. They are filled with cytoplasm with visible single dictyosomes, plastids with starch grains, lipid droplets (compatible with previous cytochemical observations), ER, and small vacuoles and mitochondria (Fig. 4h, i). All cells of the female gametophyte are connected by the plasmodesmata (Fig. 4f, g, i).

The largest accumulation of storage materials was observed in a mature, fully differentiated embryo sac (Fig. 5a). In all cells of the gametophyte, that is, synergids, egg cell, and central cell, the presence of proteins, insoluble polysaccharides, and lipids was noted. However, the central cell is distinguished by its high concentration of these compounds (Fig. 5b–d). The micropylar end of *Sedum* synergids had a filiform apparatus that stained intensely with PAS and ABB, suggesting the occurrence of polysaccharides and proteins (Fig. 5b, c).

In a mature embryo sac, the antipodes begin the process of degeneration. The egg cell extended slightly further from the micropylar apex than the synergids. From the chalazal end, the nucleus of an egg cell is surrounded by a small amount of cytoplasm with numerous plastids. Only some of them contained starch grains. Several small vacuoles with electron-dense material, with a protein character (it stained intensively with ABB) (Fig. 5b), occur at the micropylar part of the egg cell (Fig. 5e). In addition, mitochondria, a few profiles of ER, and dictyosomes are visible within its cytoplasm (Fig. 5e, f). An irregular layer of wall material, which shows a PAS-positive reaction after staining (Fig. 5c), separates the chalazal parts of the egg cell (Fig. 5e) and synergids (Fig. 5f) from the central cell. Both synergids are ultrastructurally similar, but their internal structures differ from that of the egg cell. Synergids are rich in organelles. The nucleus is located in the central part of the synergid (Fig. 5f). Characteristic appearance of their cytoplasm is associated with the accumulation of a large number of active dictyosomes, cisterns ER, and vesicles (Fig. 5f, g). At the micropylar end of the synergids, the filiform apparatus is visible. Wall ingrowths are surrounded with cytoplasm rich in mitochondria and vesicles derived from active dictyosomes (Fig. 5h).

At the cellular embryo sac stage, some portions of the cytoplasm are surrounded by an electron-translucent area. These macroautophagous structures are observed within the central cell, egg cell, and synergids (Fig. 5e, f). The mature female gametophyte is characterized by the presence of accumulated storage materials mainly in the central cell (Fig. 5e, i). Its cytoplasm contains vacuoles with electron-dense material (Fig. 5e), which stained intensively for proteins (Fig. 5b) and mitochondria, dictyosomes, ER, microbodies, plastids with starch grains, and lipid droplets (Fig. 5i).

## Discussion

*S. hispanicum* belongs to the Crassulaceae and order Saxifragales (Hutchinson 1973; Takhtajan 2009). According to our results, *S. hispanicum* ovules are anatropous, bitegmic, and crassinucellate, which agree with the earlier reports on Crassulaceae (Mauritzon 1933; Johri et al. 1992; Thiede and Eggli 2007). The anatropous ovule is one of the most common ovule types among angiosperms. It occurs in 75–80% of angiosperms (Lersten 2004). However, only among the Crassulaceae species, three types of nucelli occur: *Sedum*, *Crassula*, and *Kalanchoe*. These differ in terms of number of cells located above the megaspore mother cell and under the nucellar epidermis, appearance of epidermal cells of the nucellus, and degree of nucellar cell destruction by the developing female gametophyte (Mauritzon 1933). During the present study, we found that two layers of cells separated the megaspore mother cell from the nucellar epidermis. This shows that the nucellus conforms to the *Sedum*-type. In this type, the embryo sac is relatively small. The female gametophyte of *S. hispanicum* takes about one eighth of them, as it was reported for *S. fabaria* (Wojciechowicz and Samardakiewicz 1998). Nevertheless, in other species from the same genus, it was one third of the length of the nucellus: *S. hillebrandii*, *S. crassipes*, and *S. pachyphyllum* (Mauritzon 1933).

The megaspore mother cell of *S. hispanicum* initially slightly differs from the surrounding nucellar cells. They have a similar size and shape. However, the megasporocyte stands out against the neighboring cells with a dense cytoplasm, in which small vacuoles and an elongated nucleus are present. At this stage, in other species of angiosperms, the following have been observed in various studies: bean-shaped nucleus, which after treatment with toluidine blue does not stain in *Eleocharis sellowiana* (Rocha et al. 2015); nucleus, which changes its position during maturation of megasporocyte in *Glycine max* (Kennell and Horner 1985); and *Cymbidium sinense* (Tung et al. 1999). The nucleus of the megaspore mother cell is always very conspicuous (Bajon et al. 1999; Papini et al. 2011; Mendes et al. 2014). During development, the megasporocyte undergoes further changes, which was also reported for *Arabidopsis thaliana* (Drews and Koltunow 2011). A clearly visible feature in *S. hispanicum* is the pronounced elongation of the megaspore mother cell, whose cytoplasm becomes simultaneously less electron-dense. Cell growth is not caused by increasing vacuolar size, similar to *C. sinense* (Tung et al. 1999). The nucleus is centrally located in the megaspore mother cell of *S. hispanicum* prior to the first meiotic division. During division, the organelles are spread more or less evenly over the micropylar and chalazal ends of the cell. These observations support the conclusion that polarization is not necessary for the occurrence of meiosis (Bajon et al. 1999).

Furthermore, these observations also point to the possibility that asymmetric distribution of organelles is not maintained during the first meiotic division in *S. hispanicum*. The megaspore mother cell is characterized by a polar distribution of organelles (Tung et al. 1999; Bajon et al. 1999; Rocha et al. 2015), which is not contradicted by observations made during this study. However, it is not confirmed whether polarity is mentioned during the first and the second meiotic divisions (Yang 2006). Moreover, it was hypothesized that polarity of the megasporocyte plays a role in the selection of the functional megaspore (Willemse and Van Went 1984; Reiser and Fischer 1993). Polarization, where organelles are mostly positioned at the chalazal end of the megaspore mother cell, contribute to the fact that the chalazal functional megaspore received a significant part of the organelles (Stewart and Gifford 1967; Webb and Gunning 1990; Reiser and Fischer 1993). However, in *Eleocharis sellowiana*, the opposite polarity of the megaspore mother cell in comparison with the most common pattern described for the *Polygonum*-type is not an obstacle for the choice of the functional meiotic product (Rocha et al. 2015). The mechanisms involved in the determination of the functional megaspore have not been fully understood (Russell 2001; Qiu et al. 2008; Drews and Koltunow 2011).

The result of the second meiotic division and cytokinesis in *S. hispanicum* is one tetrad, whose megaspores are structurally similar. Multilamellar bodies (MLBs) are present in cytoplasm of all megaspores. MLBs origin is unknown. Ultrastructural analysis of tapetum in *Tillandsia* anthers revealed that multilamellar bodies can derive from plastids and/or autophagous structures (van Doorn and Papini 2016). Comparison of size, shape, and electron density of simultaneously observed plastids and MLBs in *S. hispanicum* megaspores may suggest their plastidal origin. The megaspores, compared to the megaspore mother cell, have visibly shorter cisternae of endoplasmic reticulum. These cells are always arranged linearly. This type of a tetrad is observed in *Sedum* and other members of Crassulaceae; there are also reports that within the same Crassulaceae species, two or maximum three types of megaspores occur. This has been observed in *Sedum annuum*, *S. magellense*, and *S. caeruleum* (Mauritzon 1933). In these species, linear or *T*-shaped tetrads or triads with mononuclear or binucleated micropylar cell are formed. However, the chalazal cell initiates the development of the embryo sac despite the differences in the megaspore type. In angiosperms, other configurations of megaspores have also been described in addition to the linear tetrad (Bouman 1984), for example, in *G. max* (Kennell and Horner 1985) and in *Arundo formosana* (Jane and Chiang 1996). Nevertheless, there are some exceptions, such as *Sedum sarmentosum*, *Sempervivum alpinum*, and *Chiastophyllum oppositifolium*, where sometimes a subchalazal cell becomes an embryo sac mother cell (Mauritzon 1933). Such a process does not occur in *S. hispanicum.* A female gametophyte development is *Polygonum*-type in most species of *Sedum*, as well as in the tested species. Until now, a different development path was recorded only in a few species of *Sedum*, for example, bisporic or *Allium*-type, present in *S. fabaria*, *S. populifolium*, and *S. populifolium* var. *notarjanni* (Mauritzon 1933). This type of development does not occur in other Crassulacean species.

In the walls of young megaspore mother cell of *S. hispanicum* and during its extension, simple plasmodesmata are visible, which probably allow symplasmic, direct transport of nutrients from the surrounding ovular tissues (Bajon et al. 1999; Cilia and Jackson 2004; Yang 2006). Plasmodesmata are present only in the chalazal walls in *S. hispanicum*, which simultaneously reflect the polarity of the MMC. This location is the most frequent place of their occurrence (Reiser and Fischer 1993; Bajon et al. 1999; Yang 2006). In the studied species, plasmodesmata are mentioned during megasporogenesis from the megaspore mother cell stage to the tetrad and functional megaspore stages. Such observations were also made in other angiosperms species, for example, *A. thaliana* (Bajon et al. 1999), but they can be present even at later stages, for example, *A. formosana* (Jane and Chiang 1996). Callose depositions and even the presence or absence of plasmodesmata during meiosis can temporarily regulate interactions with neighboring cells (Papini et al. 2011; Lee and Yeung 2012; Lituiev and Grossniklaus 2014). Simple plasmodesmata are observed on the walls of the surrounding nucellar cells of *S. hispanicum*.

Plasmodesmata observed during megasporogenesis in *S. hispanicum* do not have uniform but a differentiated structure. At this stage, they differ from typical and commonly visible plasmodesmata. This is the first report that describes a new kind of plasmodesmata during female gametophyte development. Simple plasmodesmata with an adjacent electron-dense dome are visible in the walls of megaspores of the tetrad and the functional megaspore from the megaspore cytoplasm side. These characteristic structures were also observed during previous ultrastructural studies conducted on this species (Kozieradzka-Kiszkurno and Bohdanowicz 2010). They were observed during *S. hispanicum* embryogenesis in suspensor cells. Compound plasmodesmata with electron-dense material from the basal cell occur between the basal cell and the first layer of the chalazal suspensor cells. Such plasmodesmata remain from the early globular embryo stage to the torpedo embryo (Kozieradzka-Kiszkurno and Bohdanowicz 2010). These types of intercellular connections have been discovered only during the process of embryogenesis of Crassulaceae species, for example, *Sedum acre*, *Sempervivum arachnoideum*, *Jovibarba sobolifera*, and *Graptopetalum bellum* (Kozieradzka-Kiszkurno and Bohdanowicz 2010; Kozieradzka-Kiszkurno et al. 2011; Kozieradzka-Kiszkurno and Płachno 2012). The place of the occurrence and shape of the unusual electron-dense material among species from the genera *Sempervivum and Jovibarba* are different from those in the genus *Sedum* (Kozieradzka-Kiszkurno et al. 2011). Such differences may occur during megasporogenesis but further studies are needed to confirm the same. Compound plasmodesmata with electron-dense dome were also observed between the basal cell and endosperm in all the aforementioned species, including *S. hispanicum* (Kozieradzka-Kiszkurno and Płachno 2012). Material adhering to the plasmodesmata from the megaspore end shows visible continuation with profiles of ER, which agree with all the earlier reports (Kozieradzka-Kiszkurno et al. 2011; Kozieradzka-Kiszkurno and Płachno 2012).

It is difficult to define the function of plasmodesmata observed in megaspore walls. Their possible function can be considered only on the basis of previous studies. Microinjection of fluorescent tracers in combination with ultrastructural studies makes it possible to increase the knowledge about the function of characteristic Crassulacean plasmodesmata during embryogenesis. Experiments conducted on *S. acre* revealed that the discovered plasmodesmata are functional, but only in directions of the basal cell-embryo proper and basal cell endosperm. Movement of symplasmic transport fluorochromes in the opposite direction was not detected, so probably they are limited (Wróbel-Marek et al. 2017). Unidirectional movement through plasmodesmata was not fully confirmed, but data revealed such a possibility (Wróbel-Marek et al. 2017). Possibly, during megasporogenesis, such plasmodesmata regulated the flow of the nutritional substances, but this is only a hypothesis. Electron-dense material visible near the plasmodesmata perhaps contributes to the results obtained; however, their chemical characters were not described. Observations made during this study do not support the possibility that electron-dense domes are callose deposits, as these structures are visible in the walls of the *S. hispanicum* functional megaspore and even at the coenocytic embryo sac stage. Callose disappears early and completely during the development of the functional megaspore (Rodkiewicz 1970; Papini et al. 2011); thus, during the aforementioned stages of development of *S. hispanicum*, the callose is theoretically no longer present. However, more cytochemical staining might verify this assumption.

Cytochemical tests in combination with ultrastructural studies allow to state precisely that proteins, lipids, and insoluble polysaccharides are present in the cells of the developing gametophyte. Cytochemical staining confirms that starch grains are accumulated within the developing female gametophyte of Crassulaceae. Similar observations were made in *S. fabaria* (Wojciechowicz and Samardakiewicz 1998) and other Crassulaceae (Maheshwari 1950; Johri et al. 1992); however, until now, it has not been cytochemically confirmed.

The megaspore mother cell is characterized by the presence of lipid droplets, starch grains, and proteins, which was also found in other species of angiosperms, such as *A. thaliana* (Bajon et al. 1999), *Tillandsia* (Papini et al. 2011), and *Paphiopedilum delenatii* (Lee and Yeung 2012). Small amounts of storage reserves were noted during megasporogenesis, but their quantity increased during megagametogenesis. As a result of successive mitotic divisions within the functional megaspore, the surrounding nucellar cells are destroyed by the growing gametophyte. This observation was also made in ovules of species from the Saxifragaceae (Wiggins 1959; Saxena 1971), which is close to Crassulaceae (Takhtajan 2009). In *S. fabaria*, the nucellar cell destruction was correlated with the accumulation of starch grains within the gametophyte (Wojciechowicz and Samardakiewicz 1998). Sporophyte is a source of nutrition for a developing gametophyte (Lituiev and Grossniklaus 2014). Moreover, plasmodesmata link all the cells of *S. hispanicum* embryo sac. Chalazal walls of the synergids and egg cell thin out and disappear locally in the mature embryo sac. This structure of a gametophyte was also observed by other authors, for example, in *A. thaliana* (Mansfield et al. 1991), *Passiflora caerulea* (García et al. 2003), and *Victoria cruziana* (Zini et al. 2016). It probably facilitates the flow of the nutritional substances and communication between individual cells of the embryo sac (Willemse and Van Went 1984; Yang 2006; Punwani and Drews 2008).

Studies conducted on *Tillandsia meridionalis* and *T. ixioides* (Bromeliaceae) ovules have revealed that degeneration of nucellar cells was caused by programmed cell death (PCD). The ultrastructural analysis of nucellar cells located around the developing female gametophyte has showed characteristic features for PCD (Brighigna et al. 2006). Some of these features were also visible in *S. hispanicum*: protoplasmic shrinkage, loss of shape of the degenerating cells, and occurrence of organelles like mitochondria, numerous small vacuoles, and a nucleus with increased electron density and enlarged perinuclear space. Described features can be an argument for the PCD (not for passive destruction). However, fragmentation of the DNA in *S. hispanicum* cells has not yet been confirmed. Early nucellar cell death of *Tillandsia* species (epiphytes) and *S. hispanicum* (leaf succulent) during female gametophyte formation may be associated with their life strategy––this aspect was also considered by Brighigna et al. (2006).

Three antipodes of *S. hispanicum* are located at the chalazal pole of the embryo sac. Each cell is mononuclear as in most members of *Sedum* (Mauritzon 1933). It is only in *S. anopetalum*, occasionally, the situation where three antipodal nuclei were not found to be separated from each other by the cell wall (Mauritzon 1933). Moreover, some antipodal cells of *S. fabaria* formed a several-celled structure called an antipodal embryo (Wojciechowicz and Samardakiewicz 1998). Arrangement of the antipodes can be different in one species, which has been shown in studies conducted on *S. acre* (Souéges 1927). Ultrastructural analysis of the megagametogenesis of *S. hispanicum* reveals that ephemeral antipodes are active cells. In some species, such as in *Aconitum vulparia* (Bohdanowicz and Turała-Szybowska 1985) and *A. thaliana* (Song et al. 2014), antipodal cells are persistent, so it appears longer during the development. In *A. vulparia*, wall ingrowths, which increase the surface for substances’ absorption, were additionally present. These structures were not observed in *S. hispanicum*, but the position of the antipodes close to the end of the funicular vascular strands indicates their nutritive function (Bohdanowicz and Turała-Szybowska 1985). Antipodal cells of *S. fabaria* also form haustoria (Mauritzon 1933; Wojciechowicz and Samardakiewicz 1998), which are engaged in the absorption of substances from surrounding cells (Johri and Ambegaokar 1984). This fact supports the possibility that *S. hispanicum* antipodal cells participate in embryo sac nutrition. However, ephemeral antipodes are also present in many other angiosperms, such as *Amaranthus hypochondriacus* (Coimbra and Salema 1999), *P. caerulea* (Garcia et al. 2003), *Cytisus multiflorus*, and *Cytisus striatus* (Rodríguez-Riaño et al. 2006). By-products of its degeneration can be used by the gametophyte (Yang 2006; Rocha et al. 2015). Degeneration of the *S. hispanicum* antipodes takes place quickly, which is similar to what has been observed in *A. hypochondriacus*. When antipodal cells started degenerating, egg apparatus of *A. hypochondriacus* were not fully mature (Coimbra and Salema 1999).

Ultrastructure of *S. hispanicum* synergids also changes after antipodal degeneration. Clearly, they become rich in organelles, particularly in active dictyosomes, which may also be involved in the formation of wall ingrowths (Coimbra and Salema 1999). Filiform apparatus is formed in the micropylar part of the cell. It increases the surface of the plasma membrane. Synergids have characteristic features of transfer cells, which promote the translocation of substances (Gunning and Pate 1969). Some substances can be absorbed and secreted. This fact is supported by the presence of mitochondria, which are in close contact with wall ingrowths. Mitochondria probably provide the energy used during substances’ absorption (Willemse and Van Went 1984). However, occurrence of numerous dictyosome vesicles near the filiform apparatus suggests that it can also be a site of substances’ secretion (Punwani and Drews 2008). The structure of filiform apparatus can be different in other species of angiosperm (Mendes et al. 2014; Zini et al. 2016). In *S. hispanicum*, it is electron-translucent, which was reported earlier in Passifloraceae (García et al. 2003) and Nymphaeaceae (Zini et al. 2016). Moreover, filiform apparatus stains intensively with ABB and PAS method, which is also commonly observed (Willemse and Van Went 1984). Moreover, synergids of *S. hispanicum* are ultrastructurally similar, which deviates from the data available for some Passifloraceae (García et al. 2003), Fabaceae (Galati et al. 2006), and Amaryllidaceae (Deng et al. 2016). It additionally points to the fact that synergids of *S. hispanicum* are not functionally differentiated.

Cytoplasm of the central cell is rich in organelles (i.e., endoplasmatic reticulum, dictyosomes, plastids, mitochondria, and microbodies), which proves that this cell is also metabolically active. The central cell dyed intensively with ABB, SBB, and PAS method. Cytochemical analyses indicate that the greatest accumulation of proteins, starch grains, and lipid droplets occur in the central cell. These investigations are consistent with previous observations, which point to high activity of the central cell (Schulz and Jensen 1973; Liu et al. 2010; Zini et al. 2016). Central cell can be engaged in the nutrition of *S. hispanicum* egg cell. This hypothesis is supported by the interrupted and thinning wall of the egg cell at the chalazal end, which was observed in *Capsella bursa-pastoris* (Schulz and Jensen 1973). The central cell can transport nutritive substances toward the egg cell (Liu et al. 2010).

Macroautophagy phenomena were observed in cells of developing female gametophyte in *S. hispanicum*. In plants, it is one of describing autophagy type next to plant microautophagy and mega-autophagy (van Doorn and Papini 2013). Moreover, plastids are possibly involved in cytoplasm degradation (plastidal autophagy) (van Doorn and Papini 2013; Papini et al. 2014). Papini et al. (2014) suggest that the autophagic processes contribute to cell reorganization during their development (e.g., in tapetal cells). This is presumably related with cytoplasm remodeling, including recycle of the organelles and cytoskeleton components (Papini et al. 2014). Macroautophagy may also play this function in *S. hispanicum* embryo sac cells.

## Conclusions

The female gametophyte of *S. hispanicum* development is of the monosporic or *Polygonum*-type. Its ovule is anatropous, crassinucellate, and bitegmic. During megasporogenesis, simple plasmodesmata with electron-dense dome occur in megaspores and coenocytic embryo sac walls. Such observations are made for the first time at this stage of development. Probably, these structures participate in the regulation of the substances’ flow, but this needs confirmation. This is the first report that describes a new kind of plasmodesmata during the development of female gametophyte.

Initially, the female gametophyte consists of seven cells, but antipodes mature rapidly during the development of the embryo sac and begin the process of degeneration. Finally, the gametophyte is built of a female germ unit (egg apparatus and central cell). Synergid cells become more active during differentiation. The central cell shows the highest concentration of the substance such as lipids, insoluble polysaccharides, and proteins after the disappearance of antipodes. To the best of our knowledge, this is the first study to reveal the ultrastructure and cytochemistry of individual cells of the Crassulaceae female gametophyte. Our results revealed many interesting features, especially during megasporogenesis. This study precisely describes the embryological development of *S. hispanicum*, which will be useful in future studies. The collected data provide basic knowledge of taxonomical value and enable further exact comparative analysis on other species from the genus *Sedum* and the family Crassulaceae.
